# Superiority of Dynamic Stretching over Static and Combined Stretching Protocols for Repeated Sprint Performance in Elite Male Soccer Players

**DOI:** 10.3390/sports13080275

**Published:** 2025-08-18

**Authors:** Ridha Aouadi, Mohamed Amine Ltifi, Halil İbrahim Ceylan, Mohamed Chedly Jlid, Nicola Luigi Bragazzi

**Affiliations:** 1 Higher Institute of Sport and Physical Education of Ksar Saîd, Research Laboratory (LR23JS01) «Sport Performance, Health & Society», Manouba University, Manouba 2010, Tunisia; ridha_aouadi@yahoo.fr (R.A.); mohamedltifi19@gmail.com (M.A.L.); chedly3@yahoo.fr (M.C.J.); 2 Higher Institute of Sport and Physical Education of Gafsa, University of Gafsa, Gafsa 2112, Tunisia; 3 Department of Physical Education of Sports Teaching, Faculty of Sports Sciences, Atatürk University, 25240 Erzurum, Turkey; halil.ibrahimceylan60@gmail.com; 4 Laboratory for Industrial and Applied Mathematics (LIAM), Department of Mathematics and Statistics, York University, Toronto, ON M3J 1P3, Canada

**Keywords:** dynamic stretching, static stretching, repeated sprint test, soccer players, injury prevention, public health promotion, warm-up protocols

## Abstract

This study aimed to examine the effects of different stretching techniques on repeated sprint performance and to assess the influence of the sequence in which static and dynamic stretching are performed. Ten male Division II soccer players (age: 22.80 ± 1.13 years; height: 180.60 ± 3.59 cm; body mass: 70.60 ± 6.04 kg) completed a repeated sprint test consisting of 6 × 30 m sprints after five different warm-up protocols in a randomized, counterbalanced design: (1) general warm-up without stretching (NS), (2) static stretching (SS), (3) dynamic stretching (DS), (4) SS followed by DS (SS-DS), and (5) DS followed by SS (DS-SS). Stretching was performed during the recovery periods between sprints: ~6 min for SS and DS, and ~12 min for combined protocols. Sessions were spaced 72 h apart. Performance metrics included mean sprint time, best sprint time, and total sprint time. ANOVA and Cohen’s d were used for statistical analysis. Repeated sprint test performance was significantly enhanced after DS compared to SS, DS-SS, and SS-DS (*p* = 0.042–0.002; ES = 0.31–2.26), but not significantly different from NS (*p* > 0.05). SS had a detrimental effect when compared to DS and NS (*p* < 0.05; ES = 1.86–2.26). Improvements were observed in mean sprint time and total sprint time across all six sprints (*p* = 0.042–0.006; ES = 0.31–2.26) and in best sprint time (*p* = 0.006–0.002; ES = 0.89–1.86). In conclusion, DS prior to repeated sprint test improves performance compared to SS and combined methods. NS also supports strong performance but shows a slight advantage over SS and combinations. Incorporating DS into warm-up routines is recommended to optimize sprint performance, reduce injury risk, and support athlete preparation.

## 1. Introduction

Warm-up routines are a critical component of athletic performance, significantly influencing physiological and biomechanical factors that contribute to optimal output [1,2]. A well-structured warm-up aims to enhance performance and reduce injury risk by preparing the muscles for subsequent exercise movements [3,4]. Stretching, as a key element of warm-up routines, plays a major role in improving flexibility, strength, and power output [5,6,7]. Additionally, warm-up activities increase body temperature, muscle strength and sensitivity, heart rate, and metabolic rate, all contributing to improved exercise efficiency [8]. Elevated body temperature reduces connective tissue stiffness, promoting greater flexibility and resilience, which is particularly beneficial for high-speed activities such as sprinting [9].

Repeated sprint ability is a critical physical quality in modern football, where match-deciding actions such as goals, defensive interceptions, or one-on-one situations often rely on short, explosive sprints. Research has shown that elite soccer players perform a high number of high-intensity efforts during matches, especially sprints under 30 m, which are typically clustered around key phases of play [10]. Majano et al. [11] highlighted the importance of repeated sprint ability, which includes the ability to perform repeated sprints and recover between efforts, both of which are essential for maintaining high performance during critical moments of the match. Improving repeated sprint ability is crucial for meeting the increasing physical demands of soccer and maximizing players’ effectiveness during these key moments.

While the general benefits of warm-up are well established, the impact of different stretching techniques on repeated sprint performance, particularly in a 30 m sprint test, remains less understood [6]. Existing research suggests that the type of stretching performed during warm-up can influence subsequent exercise performance with varying effects based on the stretching approach [7,12]. For example, studies have shown that dynamic stretching (DS) enhances sprint performance more effectively than static stretching (SS). Dsouza [12] indicated that DS positively affected 20 m sprint performance in amateur soccer players, while SS did not yield similar benefits. Furthermore, Gil et al. [2] reported that two sets of DS improved repeated-sprint performance in team sports, suggesting that the volume and type of stretching can significantly impact athletic output.

In contrast, SS has often been associated with a lack of effects on sprint performance. Research by Chaouachi et al. [13] found no significant improvement in repeated sprint performance when SS was included in the warm-up routine. Similarly, a systematic review by Li [14] indicated that while SS could enhance flexibility, it did not significantly improve explosive strength or sprint performance. These findings suggest that the choice of the stretching protocol can significantly influence sprint outcomes, highlighting the need for careful consideration of warm-up strategies.

However, recent studies seem to provide a more complex view regarding the effects of SS on repeated sprint and agility performance. For instance, Beckett et al. [15] demonstrated that SS alone during recovery periods may impair performance, whereas Wong et al. [16] found that short-duration SS followed by DS can improve flexibility without negatively affecting sprint or change-of-direction ability. Therefore, the type, duration, and sequencing of stretching techniques during warm-up are key factors to consider for optimizing repeated sprint performance.

Furthermore, the effects of stretching protocols may vary based on the specific athletic context. For instance, Fletcher and Jones [17] reported enhancement in sprint performance after DS compared to SS, whereas Samson et al. [18] noted that, while DS improved jump power, it did not significantly enhance repeated sprint performance.

Despite the growing body of research, only a limited number of studies have explored the impact of stretching exercises on repeated sprint tests in soccer players. Understanding the influence of different stretching techniques and the impact of the sequencing in which they are performed is crucial for optimizing physical performance. Although previous studies have investigated the isolated effects of SS or DS on repeated sprint performance, the combined and sequential application of these stretching modalities is relatively overlooked. Notably, no research has specifically compared the effects of performing SS before DS (SS + DS) versus the reverse order (DS + SS) on repeated sprint ability. Understanding the interaction between these protocols is essential, as the sequencing may influence neuromuscular readiness, muscle stiffness, and overall sprint output, key factors in team sports requiring high-intensity repeated efforts such as soccer.

This study aims to provide insights into the optimal warm-up strategies for enhancing sprint performance in male soccer players, contributing to the broader understanding of how stretching protocols can be effectively utilized in athletic training. The specific objectives are (i) to investigate how SS and DS during warm-up impact repeated sprint performance and (ii) to explore the effects of the sequencing in which SS and DS are performed in the warm-up.

We hypothesized that (i) combined SS-DS and/or DS-SS will be more beneficial than SS alone in enhancing repeated sprint performance in young elite soccer players; (ii) repeated sprint performance will be significantly slower after a warm-up with DS followed by SS compared to DS only; and (iii) warm-up with DS only or with SS followed by DS will produce similar repeated sprint performance outcomes.

## 2. Materials and Methods

### 2.1. Participants

The study involved ten male soccer players with an average age of 22.80 ± 1.13 years, height of 180.60 ± 3.59 cm, and body mass of 70.60 ± 6.04 kg (Table 1). Height was measured using a wall-mounted stadiometer (Seca 206, Seca GmbH & Co. KG, Hamburg, Germany) [19] with a precision of ±0.1 cm, and body mass was assessed with a calibrated digital scale (Tanita BC-545N, Tanita Corporation, Tokyo, Japan) [19] with an accuracy of ±0.1 kg. All measurements were taken with participants barefoot and wearing light clothing, following standard anthropometric procedures. All players were in good general health and were regular team-game (soccer) players participating in preseason training at the time of testing. Each player had at least 13 years of structured football experience, with a typical weekly schedule consisting of five training sessions and one official match at the end of the week. The researchers provided detailed explanations of the study to the players, and written consent was obtained from each participant, following both oral and written briefings about the experimental procedure, including its potential risks and benefits. Players were included in the study if they voluntarily consented to participate by signing a written agreement and were already members of the soccer club. Players were excluded if they had any ongoing musculoskeletal injuries, neuromuscular conditions, or other medical issues. All procedures received approval from the University’s Institutional Review Committee for the ethical participation of human subjects and were carried out in compliance with the Declaration of Helsinki.

The sample size was calculated using G*Power version 3.1. based on an F-test family, with a repeated-measures ANOVA within-subjects design (one group, five measurements, correlation among repeated measures = 0.5, nonsphericity correction ε = 1), assuming an effect size of f = 0.50, α = 0.05, and a statistical power (1–β) of 0.95. The analysis indicated that 9 participants would be sufficient; therefore, a total of 10 players were included to account for potential dropout.

### 2.2. Experimental Design

**Figure 1:**
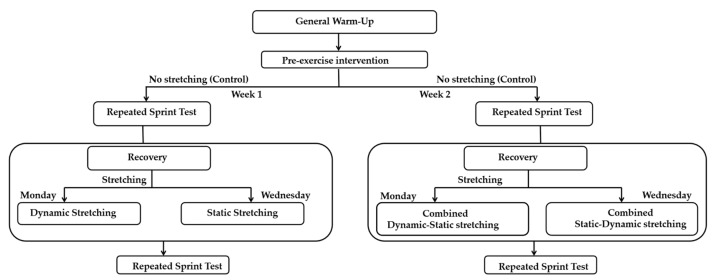
Schematic representation of the experimental protocol.

The study employed repeated sprint testing to assess performance under different stretching protocols. All participants were first familiarized with the test procedures prior to the actual testing sessions to ensure a clear understanding of the protocol. Testing was conducted over two weeks with a randomized design. Each week, participants completed sessions involving three different stretching modalities, for a total of six sessions. In the first week, the stretching protocols were: no stretching (NS), SS, and DS. In the second week, the following modalities were used: NS, combined stretching SS-DS, and combined stretching DS-SS (Figure 1).

For the combined stretching SS-DS, participants first performed SS targeting the same muscle groups as in the SS protocol, followed immediately by DS. Conversely, for the combined stretching, DS was performed first, followed by SS. A 72 h gap between testing sessions was maintained to minimize any carryover effects from the previous session.

The repeated sprint test was performed on two separate days during each week, with each session scheduled at the same time of day as the participants’ usual training sessions (evenings) to account for potential circadian rhythm influences. Testing was conducted under stable indoor conditions (23–25 °C) to ensure consistent environmental factors.

For the warm-up phase before the repeated sprint test, participants performed a series of six 30 m sprints, each at a progressively increasing speed (from approximately 70% to 100% of their maximum sprint speed), interspersed with four sets of 20 s dynamic leg swings, which gradually increased in intensity (approximately 50% to 90%, based on participant control). Participants were allowed to jog back between each set of leg swings at their own pace. After completing the assigned warm-up protocol, participants engaged in 5 min of active recovery by walking back and forth along a 30 m linear circuit.

The repeated sprint test consisted of six sets of six maximal 30 m sprints, with each sprint separated by 25 s. After each sprint, participants performed jog-back recoveries on a non-slip wooden floor inside a gymnasium. Between sets of sprints, participants had a 6 min active recovery period, consisting of:

- 1 min of standing,
- 1 min of light jogging,
- 1 min of walking around a 20 × 20 m grid, and
- 1 min of standing.

Each sprint sequence was followed by active recovery to ensure that the performance could be assessed under optimal conditions.

**Figure 2:**
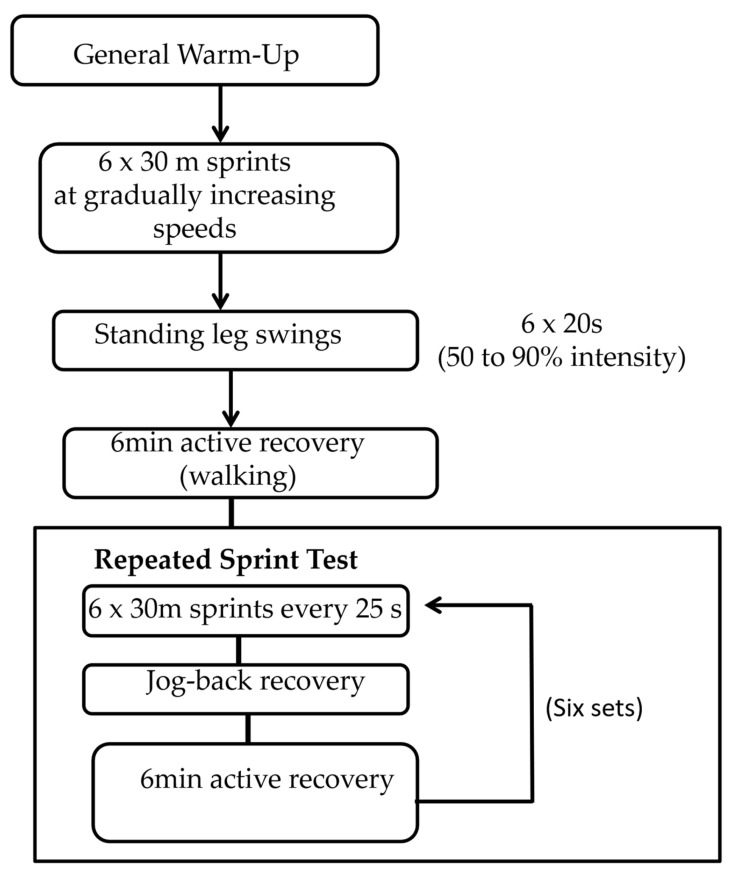
Warm-up protocol prior to repeated sprint test.

The tests were carried out across two days, with a 72 h gap between sessions. The repeated sprint performance in the NS condition acted as the control. The study was conducted in March, during the official competitive season. To reduce the impact of circadian rhythm on performance, all sessions were scheduled at the same time as the participants’ usual training sessions (in the evenings) and were conducted under stable temperature conditions (23–25 °C). The warm-up protocol is illustrated in Figure 2.

### 2.3. Training Program: General Warm Up and Stretching Protocols

The interventions were conducted following a 20 min general warm-up, which included 10 min of light jogging and a 10 min session of eight exercises (Table 2).

Following the general warm-up and pre-exercise intervention (or no intervention in the control condition), participants rested for 2 min before performing the repeated sprint test. Each stretching condition included approximately 6 min of stretching. In the control condition, the repeated sprint test was conducted after 2 min of recovery post-warm-up, without any stretching.

The SS protocol involved 6 min of lower-body stretching, followed by 5 min of walking. After walking at a moderate pace, participants performed six static stretches, repeating each stretch twice per leg (Table 3) [20]. Each stretch was held for 10 s at a mild discomfort level, followed by 5 s of relaxation before moving on to the next leg or stretch.

The stretching program was supervised by two physical education professors, the team’s head coach, and two assistant coaches. The physical trainer was responsible for demonstrating the exercises, while the other members of the staff ensured proper execution and participant safety throughout the stretching sessions.

The DS routine included ten exercises of moderate to high intensity, performed over 6 min (Table 3). Participants completed each exercise over a distance of 10 m, took a 10 s break, and then repeated the exercise while returning to the starting point. During these dynamic movements, participants were consistently guided to maintain a proper form, such as keeping the torso upright, knees aligned with the chest, and staying on their toes. Each combined stretching protocol lasted 12 min in total.

### 2.4. Repeated Sprint Performance Test

After following a standardized warm-up, which included six sub-maximal 30 m sprints, participants performed a repeated sprint performance test, which involved one set of six 30 m maximal sprints (6 × 30 m), following one of these five warm-up protocols in a within-subject, counterbalanced design: (1) a control condition with a 20 min general warm-up without stretching (NS), (2) SS, (3) DS, (4) SS followed by DS (SS-DS), and (5) DS followed by SS (DS-SS). The stretching protocols (SS, DS, SS-DS, or DS-SS) were implemented during the recovery periods between sprints. Each 30 m sprint was separated by a 6 min recovery period for the stretching protocol, except for the NS condition. The repeated sprint test performance was recorded using an electronic photocell timing system (Witty, Microgate, Bolzano, Italy), with photocells positioned at the start and end of the 30 m sprint distance. Participants began each sprint from a standing start 0.5 m behind the first gate to ensure accurate timing. The starting position was set 30 cm behind the starting photocell gate, and time measurement started when the subject traversed the first gate. Each stretching intervention lasted approximately 6 min for the SS and DS protocols and 12 min for the combined methods. The timing gates were placed 1.75 m apart, with their height set at 0.75 m. Each player started in a prone position, 0.6 m behind the starting line. Participants were instructed to sprint at their maximum effort.

### 2.5. Data Collection

Performance measures recorded included total sprint time for the six sprints in each set, mean 30 m sprint time, and best 30 m sprint time for each set, as well as the overall sprint time (sum of six sprints of each condition).

### 2.6. Statistical Analysis

One-way repeated measures analysis of variance (ANOVA) was used to assess the stretching warm-up condition-related effects. Bonferroni-adjusted pairwise post hoc comparisons were used. The effect size (ES) was determined according to Cohen’s d and classified as small (0.00 ≤ d < 0.5), medium (0.50 ≤ d < 0.8), and large (d ≥ 0.80) [21]. The assessment included reliability measures such as the intraclass correlation coefficient (ICC), standard error of measurement (SEM), SEM%, coefficient of variation (CV%), and minimal detectable difference (MDD). The SEM% was determined by dividing the SEM estimate by the participants’ means across all trials and then multiplying by 100 [22]. The CV% was calculated individually for each athlete and then averaged for the entire team by dividing the standard deviation by the mean repeated sprint test, using the formula CV = 100 × (SD/M) [23]. The interpretation of ICC values followed the guidelines of Shrout and Fleiss [24], where an ICC greater than 0.75 indicated excellent relative reliability (inter-subject variability), values between 0.40 and 0.75 were classified as fair to good, and values below 0.40 were considered poor. A CV below 10% was regarded as good reliability [25].

## 3. Results

### 3.1. Statistical Power and Reliability

The statistical power for this analysis was 0.95, with a partial eta squared value of 0.30. Furthermore, the results indicated a high level of reliability for the tests (ICC = 0.98; average CV = 2.32%).

### 3.2. Repeated Sprint Performance

The SEM% values were relatively low, ranging between 1.2% and 2.8%, indicating a good level of measurement precision. The low SEM and MDD values suggest good measurement reliability, especially under SS. The DS condition showed the highest MDD (0.164), indicating that performance changes needed to be more substantial in sprint time to be confidently interpreted as real changes rather than measurement error. Overall, the measurement system allowed for the detection of meaningful variations, but sensitivity depended on the stretching condition, with SS offering the highest sensitivity and DS the lowest.

The results in Table 4 present a clearer picture of the effects of different stretching protocols on repeated sprint performance. DS appeared to significantly improve performance across all metrics (mean sprint time, total sprint time, and best sprint time) compared to NS. However, SS, DS followed by SS, and SS followed by DS all showed significantly worse performance compared to DS alone. This seems to suggest that incorporating SS, regardless of its placement within the warm-up routine, may hinder sprint performance. The fact that SS, DS-SS, and SS-DS were also significantly worse than NS further reinforces the potential negative impact of SS on this type of activity.

Additionally, Table 5 provides a statistical comparison of repeated sprint performance following different warm-up conditions, specifically focusing on the differences between DS and other protocols (NS, SS, SS-DS, and DS-SS). For mean sprint time and total sprint time, DS showed statistically significant improvements compared to SS followed by DS, and DS followed by SS. The ES were large, particularly when comparing DS to SS, indicating a substantial difference between these conditions. There was no statistically significant difference between DS and NS for mean sprint time and total sprint time.

For best sprint time, DS again demonstrated statistically significant improvements compared to SS, SS-DS, and DS-SS, with large ESs. Similar to mean sprint time and total sprint time, there was no statistically significant difference between DS and NS for best sprint time.

The DS protocol showed superior effects on sprint performance compared to NS and SS. Compared to the SS, the DS protocol resulted in greater enhancement of mean sprint time (*p* < 0.05; ES = 2.26), total sprint time (*p* < 0.05; ES = 2.26), and best sprint time (*p* < 0.01; ES = 1.86).

### 3.3. Mean 30 m Sprint Time

The comparison of the mean 30 m sprint times across all conditions showed that the DS condition recorded the fastest mean sprint time of 4.15 ± 0.10 s, suggesting that DS provided the best overall performance outcome.

### 3.4. Total Sprint Time

The results for total sprint time during the repeated sprint ability tests are presented in Table 5. Across all trials, total sprint time generally increased from set 1 to set 6, indicating a gradual decline in sprint performance over the repeated sprints. In the DS condition, total sprint time was the fastest, demonstrating that the DS resulted in the best overall performance. The SS condition resulted in a significantly slower total sprint time compared to NS condition (*p* < 0.01), indicating that SS negatively impacted sprint performance.

The DS condition showed that it was faster than both the SS and combined methods, suggesting that DS might slightly enhance sprint performance. The combined stretching methods, DS-SS, and SS-DS showed that total sprint time values were slower than the DS but faster than SS alone.

## 4. Discussion

This study examined the acute effects of five different warm-up protocols (NS, SS, DS, SS-DS, and DS-SS) on repeated sprint performance in elite male soccer players. The main findings revealed that DS alone significantly improved repeated sprint performance across all indicators (mean sprint time, total sprint time, and best sprint time) compared to SS and the combined stretching methods, although performance under the NS condition remained comparable to DS. Conversely, SS led to significant impairments in performance when compared to both DS and NS. Regarding the stretching sequence, SS-DS yielded better performance outcomes than SS alone, partially confirming our first hypothesis. However, the DS-SS condition resulted in lower performance than DS, thus supporting our second hypothesis that combining DS with SS in this order is less effective than DS alone. Finally, similar sprint performance outcomes were not observed between the DS and SS-DS conditions, as hypothesized, since DS produced superior results. These findings suggest that incorporating DS alone into warm-up routines is most effective for optimizing sprint performance, while the inclusion of SS particularly in combination with DS may diminish the benefits of the warm-up intervention.

Recent research has begun to explore the potential of SS as an alternative to high-intensity strength training programs, raising important questions about its relevance for optimizing athletic performance [26]. Notably, studies indicate that high-intensity or high-volume SS can promote muscle hypertrophy and enhance maximal strength, yielding adaptations comparable to those achieved through traditional resistance training [9,27]. However, our findings challenge this notion.

While the previous body of scholarly literature has suggested that SS may lead to performance decrements [12,28,29], our results demonstrate that warm-ups incorporating DS resulted in significantly faster total sprint time, maximal sprint time, mean sprint time, and best sprint time compared to SS and combined stretching methods (DS-SS and SS-DS). Additionally, while previous studies have shown that the negative effects of SS can be mitigated when followed by DS, this was not fully observed in our study. One possible explanation could be the short recovery time between the stretching and the sprint test, which may not have been sufficient to reverse the neuromuscular inhibition induced by SS. Additionally, the intensity and type of DS following SS in our protocol may not have been enough to fully counteract the acute negative effects. Finally, individual variability and training level could also influence the recovery of performance after SS. These factors may explain why no clear performance restoration was observed in the SS-DS or DS-SS conditions in our study. Nonetheless, our results specifically indicate that SS does not confer performance benefits for sprinting and may, in fact, lead to slower times, corroborating prior research that highlights its potential negative impact on sprint performance [12,29].

In agreement with Ltifi et al. [19], who examined the effects of various stretching methods on change-of-direction performance in soccer players, our study underscores the significant impact of stretching protocols on athletic performance. Ltifi et al. (2023) [19] found that DS was the most effective for improving change-of-direction performance, while SS negatively affected performance outcomes. Similarly, our results suggest that DS led to better sprint times compared to SS and combined stretching methods. This is consistent with the broader literature indicating that SS may not enhance performance and could hinder explosive movements such as sprinting or change-of-direction performance [12,26].

Regarding repeated sprint test, our findings suggest that warm-ups without stretching or those incorporating DS may be more effective. Additionally, concluding a warm-up with SS appears to be the least favorable option for the repeated sprint test, aligning with Fletcher and Jones [17] who reported slower 20 m sprint times following passive stretching compared to sprinting before stretching. The negative impact of SS on repeated sprint test, particularly in comparison to DS and the NS condition, is consistent with the existing body of scholarly literature [12,26]. While SS enhances muscle flexibility, it may also acutely decrease muscle stiffness and power output, which are crucial for explosive movements like sprinting [26]. This effect may arise from reduced muscle stiffness and disrupted neural pathways involved in muscle activation. In contrast, DS actively engages muscles through their range of motion, likely priming the neuromuscular system for sprinting demands [26].

The strong performance observed in the NS condition suggests that athletes who prefer not to stretch may benefit from skipping stretching altogether, consistent with studies indicating that shorter durations of stretching or training in highly conditioned individuals may not negatively impact performance [25]. The significantly slower sprint times following SS and combined stretching methods warrant further investigation. It is plausible that SS prior to dynamic activities interferes with neuromuscular activation and power production.

The mechanisms underlying this interaction remain unclear and merit additional exploration. Furthermore, the duration and intensity of SS protocols should be considered, as excessive SS has been shown to negatively impact performance [26]. Future research examining the relationship between range of motion and stretch-induced deficits could provide valuable insights.

Interestingly, while total sprint time did not significantly differ between DS and DS-SS, mean sprint time, total sprint time, and best sprint time were notably worse in the DS-SS condition. This suggests that, while overall accumulated time may not be drastically affected by adding SS after DS, peak sprint speed may still be compromised. Regarding the absence of an order effect in the DS and SS combination, we believe this may be attributed to the relatively short duration of each stretching protocol (6 min for SS and DS, and 12 min for the combined SS-DS and DS-SS protocols). Given the brief time spent on each type of stretching, it is possible that there was not enough time for one type of stretching to significantly influence the effectiveness of the other, as previously mentioned. Additionally, the elite condition of the athletes in our study may have mitigated any potential order effect, allowing them to adapt quickly to the different stretching sequences. This lack of an order effect might also be due to the balance of neuromuscular activation provided by both types of stretching, preventing one from interfering with the other in a way that would significantly alter performance outcomes.

Taken all together, these findings emphasize the importance of selecting appropriate warm-up protocols to optimize sprint performance. DS appears to be the most effective approach, while SS, whether alone or in combination, may be detrimental. The fact that the NS warm-up resulted in relatively strong performance, surpassing SS and combined methods, suggests that a general warm-up alone may suffice for preparing soccer players for repeated sprints. However, the slight advantage of DS over NS highlights the potential for further performance enhancement through targeted warm-up strategies.

### 4.1. Practical Implications and Future Directions

The findings of this study have practical implications for performance enhancement and injury prevention in soccer [5,7]. Incorporating DS into warm-up routines can significantly improve repeated sprint performance and may reduce injury risk by enhancing flexibility, neuromuscular activation, and muscle–tendon compliance [18,26,30]. These effects are particularly relevant in soccer, where explosive movements and rapid directional changes are frequent. Warm-up strategies that emphasize DS or NS components should be prioritized by coaches to optimize physical readiness and reduce musculoskeletal injury risk.

Moreover, integrating DS-based warm-up protocols into broader training programs, such as the FIFA 11+, has shown additional benefits for sprinting and agility while lowering injury incidence [19,31]. These findings support the implementation of structured DS routines not only in elite sports but also in school and community-based physical activity programs to promote long-term musculoskeletal health and physical independence.

However, the current findings are specific to repeated sprint performance in young male soccer players and may not be generalizable to other sports or populations. Future research should examine the effects of different stretching protocols across various age groups, athletic disciplines (e.g., basketball, track and field), and skill levels (elite vs. amateur), as athlete status may influence responsiveness [32]. Further studies should also explore the physiological mechanisms underlying the observed effects, particularly in terms of muscle stiffness, temperature, elasticity, and neural activation, to refine warm-up recommendations and optimize performance outcomes.

### 4.2. Study Limitations

This study has several limitations that may affect the generalizability of the findings. The limited sample size and the specific focus on male soccer players may restrict applicability to other athletic populations, including female athletes or those from different sports. However, the number of subjects was determined based on strict inclusion criteria (e.g., competitive level, training history, and absence of injury), which limited the pool of eligible participants. Moreover, our study was designed as a preliminary investigation or pilot study, aiming to explore the potential effects of the warm-up intervention in a controlled and homogeneous population. Despite the limited sample, appropriate statistical methods were applied, and ESs were reported to provide a meaningful interpretation of the results. Future research should aim to include larger and more diverse samples to enhance external validity.

Additionally, the study did not account for potential confounding variables such as individual flexibility levels or prior training history, which could influence the effectiveness of stretching protocols. Further investigations are needed to explore the optimal duration and intensity of DS-based protocols for improving repeated sprint performance, as well as the long-term effects of consistent DS implementation.

## 5. Conclusions

These results highlight that DS, performed before repeated sprints, leads to the best performance outcomes across multiple sprint tests (mean sprint time, total sprint time, and best sprint time), outperforming SS and combined methods. While NS also shows strong results, DS provides a more consistent advantage in enhancing sprint performance. Therefore, incorporating DS into warm-up routines is recommended to optimize athletic performance and reduce injury risk.

## Figures and Tables

**Table 1:** Mean values (Means ± SD) of age, experience, and anthropometric data in the sample of soccer players (*n* = 10).

	Mean ± SD
Age (years)	22.80 ± 1.13
Body mass (kg)	70.60 ± 6.04
Height (cm)	180.60 ± 3.59
Body Mass Index (kg/m^2^)	21.62 ± 1.32

**Table 2:** Overview of the general warm-up performed by participants.

Warm-Up Jog: Light Jogging for 10 min
Exercise 1: Gentle running for 3 min
Exercise 2: Running backward for 1 min
Exercise 3: Lateral movement for 1 min
Exercise 4: Forward walking with knee lifts to chest and a twist of the torso for 1 min
Exercise 5: Sprinting in a straight line for 5 m, repeated twice with 10 s rest intervals, lasting 1 min
Exercise 6: Sprinting in a straight line for 10 m, repeated twice with 10 s rest intervals, lasting 1 min
Exercise 7: Zigzag running for 20 m, repeated twice with 10 s rest intervals, lasting 2 min

**Table 3:** Static and dynamic stretching exercises [20].

N°	Exercise Type	Exercise Name	Image/Figure	Description
1	Static Stretching Exercices	Adductor Stretch	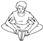	Seated with an upright spine, bring the soles of the feet together, bend the knees, and allow them to drop outward
2		Modified Hurdlers Stretch	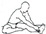	Seated with one leg straight, position the other foot against the inside of the straight leg, then reach forward toward the straight leg
3		External Hip Rotator Stretch	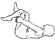	Lying down, cross one ankle over the opposite knee to form a “4” shape, then pull the uncrossed leg towards the chest, flexing the hips to around 90 degrees or more
4		Bent-over Toe Raise	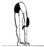	From a standing position with one foot slightly ahead of the other, dorsiflex the front foot toward the shin while leaning the upper body forward
5		Quadriceps Stretch	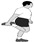	Standing straight, bend one knee to bring the heel toward the buttocks, holding the foot with one hand
6		Calf Stretch	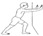	Standing with feet staggered about 2 to 3 feet from a wall, lean forward with both hands against the wall, keeping the back leg straight and the front leg slightly bent
7	Dynamic Stretching Exercices	High-Knee Walk	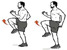	Walk forward, lifting each knee toward the chest, rising onto the toes, and swinging alternating arms
8		Straight-Leg March	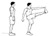	Extend both arms in front of the body while walking, lift one straight leg toward the hands, then return to the starting position and repeat with the opposite leg
9		Hand Walk	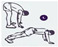	Start with hands and feet on the ground, limbs extended, walk the feet toward the hands while keeping the legs straight, then walk the hands forward, maintaining straight limbs
10		Lunge Walks	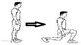	Step forward into lunges with alternating legs, keeping the torso upright throughout the movement
11		Backward Lunge	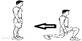	Take large steps backward with each leg alternately, aiming to reach as far back as possible
12		High-Knee Skip	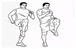	Skip forward, focusing on height, lifting the knees high, and swinging the arms actively
13		Lateral Shuffle	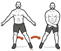	Move quickly to the side without crossing your feet
14		Back Pedal	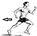	Take small, rapid steps backward while keeping the feet under the hips
15		Heel-ups	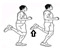	Move forward, quickly kicking the heels toward the buttocks with each step
16		High-Knee Run	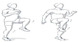	Run forward at a fast pace, emphasizing lifting the knees high and swinging the arms actively

**Table 4:** Repeated sprint performance (Means ± SD) following different stretching protocols. Mean values (Means ± SD) of the sprint speed (sprint 10 m, 20 m, and 30 m) in soccer players (N = 10) are reported.

Repeated Sprint 30 m Time (Seconds)	NS	SS	DS	DS-SS	SS-DS
Sprint 1	4.28 ± 0.16	4.33 ± 0.22	4.09 ± 0.17	4.37 ± 0.25	4.37 ± 0.16
Sprint 2	4.30 ± 0.14	4.36 ± 0.16	4.05 ± 0.25	4.36 ± 0.23	4.46 ± 0.23
Sprint 3	4.34 ± 0.28	4.42± 0.19	4.06 ± 0.18	4.39 ± 0.21	4.42 ± 0.15
Sprint 4	4.29 ± 0.21	4.43 ± 0.18	4.14 ± 0.24	4.37 ± 0.19	4.45 ± 0.18
Sprint 5	4.26 ± 0.14	4.42 ± 0.16	4.29 ± 0.21	4.41 ± 0.20	4.42 ± 0.14
Sprint 6	4.32 ± 0.21	4.39 ± 0.19	4.29 ± 0.19	4.35 ± 0.22	4.53 ± 0.19
Mean 30 m sprint time	4.30 ± 0.03	4.39 ± 0.04 *^¥^	4.15 ± 0.11 *	4.36 ± 0.02 ^¥^	4.44 ± 0.05 **^¥¥^
Total sprint time	25.79 ± 1.15	26.37 ± 1.13 **^¥^	24.92 ± 1.24	26.17 ± 1.31 ^¥^	26.66 ± 1.06 **^¥¥^
Best sprint time	25.04 ± 0.88	25.78 ± 1.39 ^¥¥^	23.69 ± 1.37	25.66 ± 1.12 ^¥¥^	26.14 ± 0.91 ^¥¥^

DS: dynamic stretching; SS: static stretching; NS: no stretching. ** significantly different (*p* < 0.01) compared to NS; * significantly different (*p* < 0.05) compared to NS. ¥¥ significantly different (*p* < 0.01) compared to DS; ¥ significantly different (*p* < 0.05) compared to DS.

**Table 5:** Repeated sprint performance following different warm-up conditions.

Performance Time	DS vs. NS		DS vs. SS		DS vs. SS-DS		DS vs. DS-SS	
	*p*	ES	*p*	ES	*P*	ES	*p*	ES
Mean Sprint Time	0.322	1.20	0.026	2.26	0.006	0.31	0.042	2.03
Total Sprint Time	0.322	1.20	0.026	2.26	0.006	0.31	0.042	2.03
Best Sprint Time	0.203	0.89	0.002	1.86	0.002	1.86	0.006	1.63

DS: dynamic stretching; ES: effect size; SS: static stretching; NS: no stretching.

## Data Availability

Information is available upon request in accordance with relevant restrictions (e.g., privacy or ethics).

## Abbreviations

The following abbreviations are used in this manuscript:

DS	Dynamic Stretching
SS	Static Stretching
NS	No Stretching
DS-SS	Dynamic Stretching followed by Static Stretching
SS-DS	Static Stretching followed by Dynamic Stretching
ES	Effect Size
ANOVA	Analysis of Variance
SD	Standard Deviation
ICC	Intraclass Correlation Coefficient
SEM	Standard Error of Measurement
SEM%	Percent of Standard Error of Measurement
CV%	Coefficient of Variation
MDD	Minimal Detectable Difference

## References

[1] Fradkin A.J., Zazryn T.R., Smoliga J.M. (2010). Effects of warming-up on physical performance: A systematic review with meta-analysis. J. Strength. Cond. Res..

[2] Gil M.H., Neiva H.P., Sousa A.C., Marques M.C., Marinho D.A. (2019). Current Approaches on Warming up for Sports Performance: A Critical Review. Strength Cond. J..

[3] Cirino C., Marostegan A.B., Hartz C.S., Moreno M.A., Gobatto C.A., Manchado-Gobatto F.B. (2023). Effects of Inspiratory Muscle Warm-Up on Physical Exercise: A Systematic Review. Biology.

[4] Ribeiro B., Pereira A., Neves P., Marinho D., Marques M., Neiva H.P. (2021). The effect of warm-up in resistance training and strength performance: A systematic review. Motricidade.

[5] Warneke K., Wohlann T., Lohmann L.H., Wirth K., Schiemann S. (2023). Acute effects of long-lasting stretching and strength training on maximal strength and flexibility in the calf muscle. Ger. J. Exerc. Sport Res..

[6] Behm D.G., Blazevich A.J., Kay A.D., McHugh M. (2016). Acute effects of muscle stretching on physical performance, range of motion, and injury incidence in healthy active individuals: A systematic review. Appl. Physiol. Nutr. Metab..

[7] Peck E., Chomko G., Gaz D.V., Farrell A.M. (2014). The effects of stretching on performance. Curr. Sports Med. Rep..

[8] Kyranoudis A.E., Ispyrlidis I., Chatzinikolaou A., Gargalianos D., Michailidis Y., Papadopoulou S.D., Kyranoudis E., Metaxas T. (2021). Effect of the pre-warm-up exercise program on muscle performance. J. Phys. Educ. Sport..

[9] Marinho D.A., Gil M.H., Cardoso Marques M., Barbosa T.M., Neiva H.P. (2017). Complementing Warm-up with Stretching Routines: Effects in Sprint Performance. Sports Med. Int. Open.

[10] Mohr M., Krustrup P., Bangsbo J. (2003). Match performance of high-standard soccer players with special reference to development of fatigue. J. Sports Sci..

[11] Majano C., García-Unanue J., Hernandez-Martin A., Sánchez-Sánchez J., Gallardo L., Felipe J.L. (2023). Relationship between Repeated Sprint Ability, Countermovement Jump and Thermography in Elite Football Players. Sensors.

[12] Dsouza C.D., Kumar N. (2024). A study to evaluate the immediate effect of proprioceptive neuromuscular facilitation versus active dynamic stretching during warm-up on 20-meter sprint in amateur soccer players. Int. J. Res. Med. Sci..

[13] Chaouachi A., Castagna C., Chtara M., Brughelli M., Turki O., Galy O., Chamari K., Behm D.G. (2010). Effect of warm-ups involving static or dynamic stretching on agility, sprinting, and jumping performance in trained individuals. J. Strength. Cond. Res..

[14] Li Y. (2023). The effects of static stretching on explosive strength and sprint performance: A systematic review. J. Strength. Cond. Res..

[15] Beckett J.R., Schneiker K.T., Wallman K.E., Dawson B.T., Guelfi K.J. (2009). Effects of static stretching on repeated sprint and change of direction performance. Med. Sci. Sports Exerc..

[16] Wong D.P., Chaouachi A., Lau P.W., Behm D.G. (2011). Short Durations of Static Stretching when Combined with Dynamic Stretching do not Impair Repeated Sprints and Agility. J. Sports Sci. Med..

[17] Fletcher I.M., Jones B. (2004). The effect of different warm-up stretch protocols on 20 meter sprint performance in trained rugby union players. J. Strength. Cond. Res..

[18] Samson M., Button D.C., Chaouachi A., Behm D.G. (2012). Effects of dynamic and static stretching within general and activity specific warm-up protocols. J. Sports Sci. Med..

[19] Ltifi M.A., Jlid M.C., Coquart J., Maffulli N., van den Tillaar R., Aouadi R. (2023). Acute Effect of Four Stretching Protocols on Change of Direction in U-17 Male Soccer Players. Sports.

[20] Faigenbaum A.D., Bellucci M., Bernieri A., Bakker B., Hoorens K. (2005). Acute effects of different warm-up protocols on fitness performance in children. J. Strength. Cond. Res..

[21] Cohen J. (1998). Statistical Power Analysis for the Behavioral Sciences.

[22] Pérez-Ifrán P., Rial M., Brini S., Calleja-González J., Del Rosso S., Boullosa D., Benítez-Flores S. (2022). Change of Direction Performance and its Physical Determinants Among Young Basketball Male Players. J. Hum. Kinet..

[23] Macadam P., Cronin J.B., Feser E.H. (2022). Acute and longitudinal effects of weighted vest training on sprint-running performance: A systematic review. Sports Biomech..

[24] Shrout P.E., Fleiss J.L. (1979). Intraclass correlations: Uses in assessing rater reliability. Psychol. Bull..

[25] Brughelli M., Van Leemputte M. (2013). Reliability of power output during eccentric sprint cycling. J. Strength. Cond. Res..

[26] Behm D.G., Chaouachi A. (2011). A review of the acute effects of static and dynamic stretching on performance. Eur. J. Appl. Physiol..

[27] Asgari M., Nazari B., Bizzini M., Jaitner T. (2023). Effects of the FIFA 11+ program on performance, biomechanical measures, and physiological responses: A systematic review. J. Sport. Health Sci..

[28] Ishak A., Ahmad H., Wong F.Y., Rejeb A., Hashim H.A., Pullinger S.A. (2019). Two Sets of Dynamic Stretching of the Lower Body Musculature Improves Linear Repeated-Sprint Performance in Team-Sports. Asian J. Sports Med..

[29] Yanci J., Iturri J., Castillo D., Pardeiro M., Nakamura F.Y. (2019). Influence of warm-up duration on perceived exertion and subsequent physical performance of soccer players. Biol. Sport..

[30] Opplert J., Babault N. (2018). Acute Effects of Dynamic Stretching on Muscle Flexibility and Performance: An Analysis of the Current Literature. Sports Med..

[31] Foqha B.M., Schwesig R., Ltifi M.A., Bartels T., Hermassi S., Aouadi R. (2023). A 10-week FIFA 11+ program improves the short-sprint and modified agility T-test performance in elite seven-a-side soccer players. Front. Physiol..

[32] Konrad A., Močnik R., Nakamura M., Sudi K., Tilp M. (2020). The Impact of a Single Stretching Session on Running Performance and Running Economy: A Scoping Review. Front. Physiol..

